# Thermally Tunable Structural Coloration of Water/Surfactant/Oil Emulsions

**DOI:** 10.1021/acs.langmuir.1c03020

**Published:** 2021-12-22

**Authors:** Yuto Arai, Nayuta Yashiro, Yoshiro Imura, Ke-Hsuan Wang, Takeshi Kawai

**Affiliations:** Department of Industrial Chemistry, Tokyo University of Science, 1-3 Kagurazaka, Shinjuku-ku, Tokyo 162-8601, Japan

## Abstract

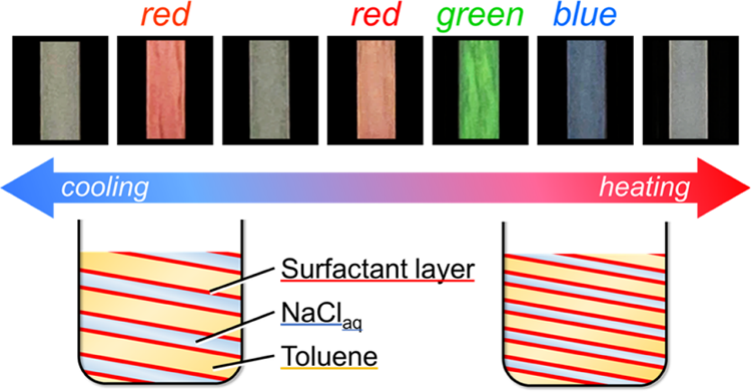

Stimuli-responsive structural color in nature has fascinated scientists, directing them to develop artificial coloration materials that adjust colors in response to external stimuli. Many stimuli-responsive structural color materials have been realized. However, only a few have reported on all-liquid-type materials, which have a particularly desirable feature because they impart their function to the device of any shape. We have previously reported the development of a consistent structural color within a narrow temperature range for all-liquid-type emulsions comprising a long-chain amidoamine derivative (C18AA) and tetraoctylammonium bromide (TOAB). In the present study, we demonstrate that introducing NaCl as an electrolyte affords a highly thermo-sensitive color-changing ability to the emulsions. The structural color of the emulsions can be controlled from red to blue by tuning the temperature. Furthermore, the C18AA and TOAB concentrations can independently regulate the color and coloring-temperature, respectively, realizing that the desired color can develop at a given temperature.

## 1 Introduction

Various plants and animals in nature exhibit an array of structural colors, such as the brilliant blue of the *Morpho* butterflies, iridescent green of some beetles, and iridescent eye-like pattern of peacock tail feathers, attracting the attention of many researchers.^1−5^ Consequently, artificial materials mimicking the structural colors in nature were developed and many efforts were devoted to constructing photonic materials with various nanostructures.^1−9^ Generally, the fabrication methods are categorized into top-down and bottom-up strategies. The top-down technique produces a wide range of high-quality photonic nanopatterns; however, this technique is expensive and tedious and has resolution limitations of approximately 100 nm.^10,11^ The bottom-up technique utilizes basic building blocks, such as colloidal particles, through self-assembly processes to construct periodic ordered nanostructures that exhibit structural colors.^11,12^

Furthermore, some animals adjust their structural colors depending on their environment,^1−4^ which inspired several scientists to develop artificial structural color materials responding to external stimuli.^1−4,8,9^ There are many reports on materials with tunable structural colors depending on the surrounding stimuli, such as temperature,^13−17^ pH,^18−20^ magnetic field,^21−26^ electric field,^24,27,28^ humidity,^29−33^ and mechanical stress.^34−38^ Most structural color materials consist of periodic arrayed objects and media; their color tuning mainly relies on changing the periodic spacing of the objects or the refractive index contrast between the objects and the media. For example, adjusting the interparticle separation with an external magnetic field can control the structural color of the colloidal particles containing magnetic nanocrystals.^21^ Yin et al. reported the reversible color changes with varying relative humidities in polystyrene colloids immobilized on polyacrylamide hydrogels.^29^ In addition, Lee et al. demonstrated the electrical tunability of the dispersion color of Fe_3_O_4_@SiO_2_ core–shell particles embedded between indium tin oxide electrodes.^24^

Objects and media composed of structural color materials, in particular variable structural color materials, are exclusively solid–liquid or solid–solid combinations; however, there are very few all-liquid-type materials.^39,40^ Liquid-type structural color materials are desirable because they can impart their function to the device of any shape by pouring them into the container of the device. The dispersion of a polymer and SiO_2_ colloid particles is a liquid-type coloring material;^22−24^ however, it is necessary to improve the dispersion stability of their solid particles and prevent their sedimentation. Some aqueous surfactant solutions present the features of all-liquid-type coloring materials.^39,40^ Nevertheless, their solution color is adjustable by the system composition, such as the surfactant concentration, and not by external stimuli. As an adjustable all-liquid-type structural color system, Cong et al. successfully demonstrated the development of the iridescent color by a self-assembled nonionic surfactant in water, which is tunable by temperature.^40^ The iridescent color, however, did not have a high thermal sensitivity, thereby requiring an increase in the temperature of approximately 25 °C to observe the color change (e.g., from blue to green).

**Figure 1:**
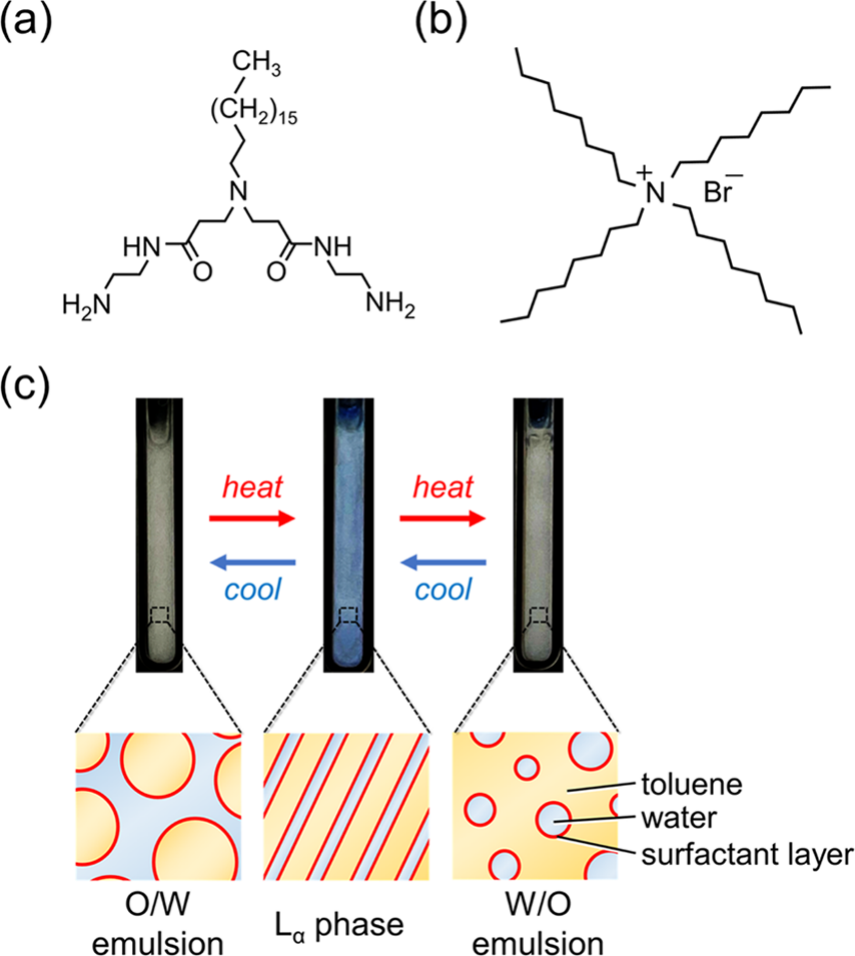
Molecular structure of (a) C18AA and (b) TOAB. (c) Schematic illustration of the phase inversion of iridescent emulsions during the coloration process.

Our previous study reported an all-liquid-type coloring emulsion comprising a long-chain amidoamine derivative (C18AA; Figure 1a) and tetraoctylammonium bromide (TOAB; Figure 1b).^41^ The emulsion demonstrated an oil-in-water (O/W) to water-in-oil (W/O) phase inversion upon heating, passing through a lamellar phase that developed the iridescent color. The coloration was attributed to the optical interference generated from the periodic lamellar structure composed of water, toluene, and surfactant layers of C18AA and TOAB (Figure 1c). The color developed in a specific narrow temperature range (approximately 3 °C) was controlled by adjusting the C18AA concentrations; however, the developed color was a constant tone in the whole coloring-temperature range and was incapable of tuning by the external stimuli. In this study, we demonstrate that the addition of NaCl as an electrolyte to the C18AA and TOAB coloring emulsions generates two coloring-temperature regions and imparts an adjustable ability of structural color to the emulsions. The emulsions in the upper coloring-temperature region developed a thermosensitive color. In particular, the color of the emulsion showed a blue shift upon heating, which was highly sensitive to temperature and could be changed from red to blue only by raising the temperature to a few degrees. The emulsion color in the lower coloring-temperature region was constant, and the color was controllable by the C18AA concentration. Furthermore, the NaCl and TOAB concentrations regulated the coloring-temperature.

## 2 Experimental Section

All chemicals were of reagent grade and were obtained from Sigma-Aldrich (St. Louis, USA), Kanto Chemical Co., Inc. (Tokyo, Japan), and Tokyo Chemical Industry (Tokyo, Japan). Commercially available reagents and solvents were used without further purification, except for methyl acrylate, which was purified by distillation under reduced pressure. (*N*-(2-Amino-ethyl)-3-{[2-(2-amino-ethylcarbamoyl)-ethyl]-octadecyl-amino}-propioamide (C18AA, Figure 1a) was synthesized according to a previously reported procedure.^42^

A typical iridescent emulsion containing an electrolyte was prepared as follows: a toluene solution of TOAB (1.5 mL) was added to an aqueous solution of C18AA (0.2 mL) and an electrolyte; the mixture was sonicated for 5 min (20 kHz, 50 W). The total concentrations of C18AA, TOAB, and the electrolyte were 25, 11.4, and 1 mM, respectively. The volume fraction of toluene was fixed at 0.88 for all the experiments, whereas the concentrations of C18AA, TOAB, the electrolyte, and the electrolyte species were varied for preparing various iridescent emulsions.

The direct reflection spectra of C18AA + TOAB emulsions were recorded using a UV–visible spectrometer (JASCO, V570, Japan) equipped with an absolute reflectivity accessory (JASCO, ARN-475, Japan) at the interface of a quartz cell filled with the emulsion. The lattice spacing of the iridescent emulsion periodic structure was calculated using the Bragg–Snell equation (eq 1).
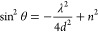
1where λ, *d*, θ, and *n* are the reflection peak wavelength, lattice spacing, incident angle, and average refractive index of the emulsion, respectively. An optical microscope (Leica Microsystems, DM2500M, Germany) was used for microscopic observations. The temperature of the samples was controlled using a thermostatic water jacket maintained by a refrigerated bath circulator.

## 3 Results and Discussion

### 3.1 Effect of Electrolyte Introduction on Coloring Emulsions

**Figure 2:**
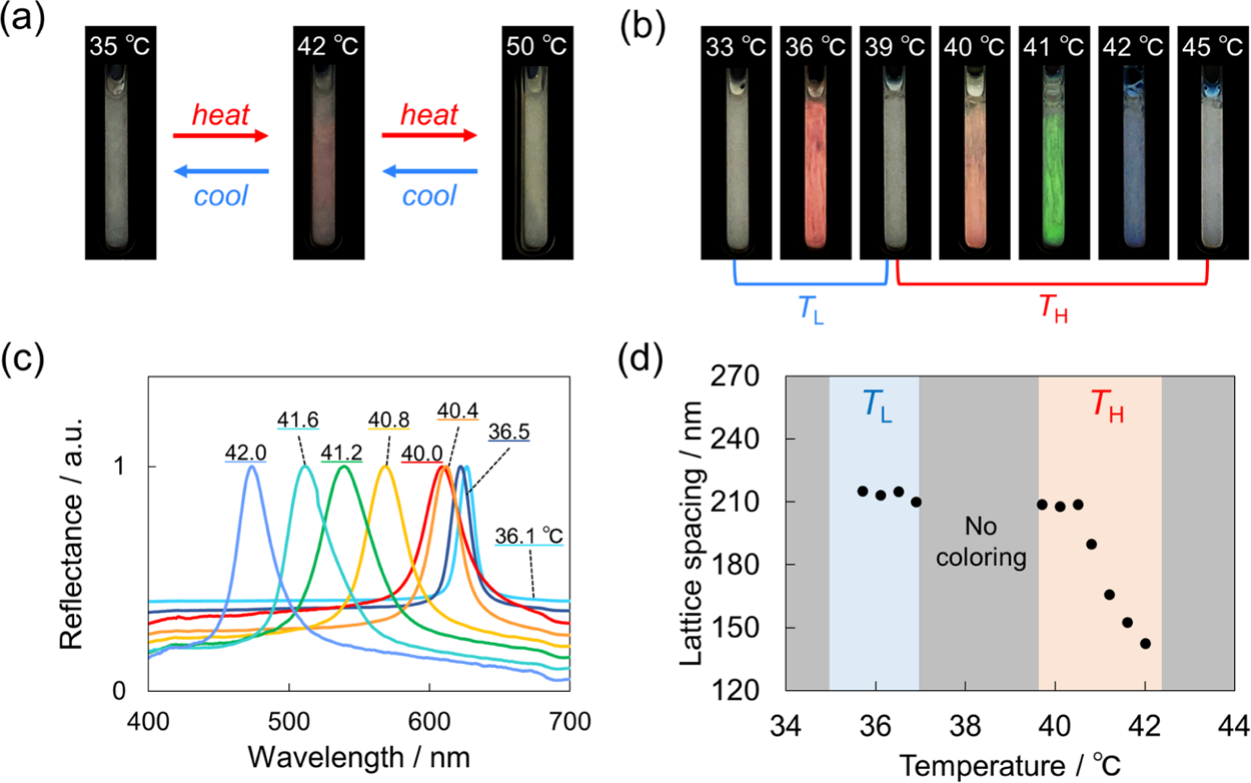
Photographs of an (a) original iridescent emulsion without NaCl and (b) iridescent emulsion with 1 mM NaCl. (c) Direct ultraviolet–visible reflection spectra at an incident angle of 15° and (d) temperature dependence of lattice spacing of emulsions with 1 mM NaCl. All samples had [C18AA] = 25 mM and [TOAB] = 11.4 mM.

Our previous paper reported that the C18AA and TOAB emulsions in water and toluene mixtures were transformed from the O/W to the W/O emulsion phase through a bicontinuous lamellar phase upon heating. The lamellar phase developed an interference structural color derived from a periodic layered structure of water and toluene, where the C18AA and TOAB molecules were adsorbed on the interface between water and toluene.^41^ Although the emulsions developed a constant color in a single coloring-temperature region, the addition of NaCl to the emulsions resulted in the appearance of two coloring-temperature regions and incorporation of a distinguishing thermosensitive color-changing feature. For example, the original emulsions of C18AA and TOAB with 25 and 11.4 mM concentrations, respectively, and without NaCl showed a pale red color in the temperature range of 40.9–42.9 °C (Figure 2a). However, the emulsions containing an aqueous solution of 1 mM NaCl had two coloring-temperature regions of 34.8–36.9 and 39.6–42.6 °C and developed a thermal variable color at 40.5–42.6 °C (Figure 2b). Although the iridescent color of the emulsions containing NaCl was retained in the lower coloring-temperature region (*T*_L_), the color in the higher coloring-temperature region (*T*_H_) was thermally sensitive and varied from red to blue upon heating. To the best of our knowledge, the high thermal variability of color is an uncommon phenomenon in liquid-type structural color systems; thus, this is an important finding for developing tunable structural color materials based on all-liquid-type compositions. Further, the iridescent emulsions with the high thermal variability of color were stable for at least a month.

Because the iridescent color in both temperature regions was derived from an optical interference of the periodic layered structure, the specular reflection spectra of the emulsions were measured at various temperatures to ascertain this expectation. A strong reflection peak appeared in each reflection spectrum of the emulsions in both coloring-temperature regions (Figure 2c). The reflection color was changed from red to blue, and the reflection peak shifted toward a shorter wavelength with an increasing incident angle of light (Figure S1a,b), a typical interference color characteristic. Furthermore, λ^2^ was plotted against sin^2^ θ because the interference color satisfies the Bragg–Snell equation. The linear relationship of the plots in Figure S1c indicates that the iridescent color derives from the interference; hence, the periodic layered structures in the emulsions lead to an iridescent color in both the coloring-temperature regions.

Figure 2d shows the *d* of the periodic layered structure that is evaluated from the Bragg–Snell equation. The absence of plots in the temperature range of 36.9–39.6 °C implied the absence of a reflection peak, suggesting the disappearance of the periodic layered structure. The constant *d* value of 210 nm in the *T*_L_ region was consistent with retaining the red color in the visual observations. However, the value rapidly decreased with increasing temperature in the *T*_H_ region above 40.5 °C, as shown in Figure 2b, which also demonstrates the gradual color change from red to blue upon heating. Interestingly, the *d* value in the *T*_H_ region at lower temperatures is practically identical to that in the *T*_L_ region. Consequently, we successfully demonstrated that introducing NaCl generates a highly thermo-sensitive color-changing ability in the C18AA and TOAB emulsions. Thus, increasing the temperature by a few degrees can change the emulsion color from red to blue.

**Figure 3:**
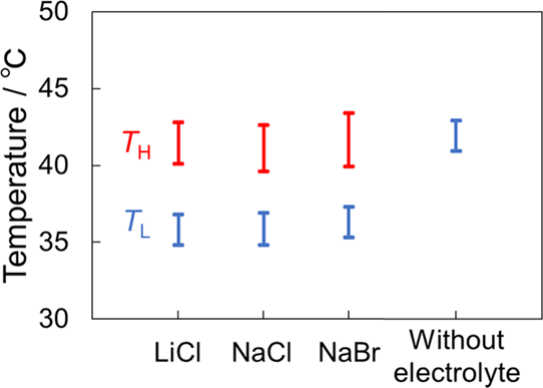
Effect of the electrolyte type on the coloring-temperature regions at [C18AA] = 25 mM and [TOAB] = 11.4 mM. The concentration of the electrolytes was 1 mM.

Because NaCl strongly influenced the coloring behavior of the emulsions owing to the electrolyte effect, other electrolytes were also examined. The aqueous solutions of LiCl (1 mM) and NaBr (1 mM) demonstrated a similar coloring behavior; their emulsions had two coloring-temperature regions (Figure 3), with a red color in the in *T*_L_ region that changed to blue in the *T*_H_ region upon heating (Figure S2). Furthermore, the temperature range was independent of the electrolyte type.

### 3.2 Effect of the NaCl Concentration

**Figure 4:**
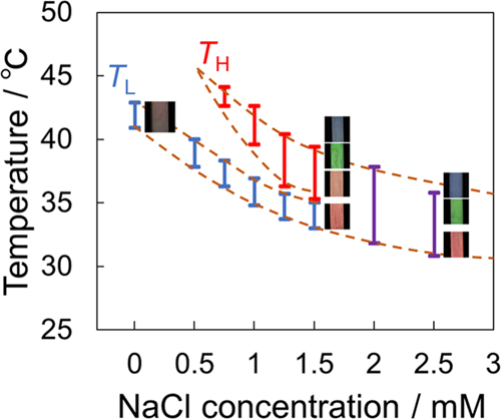
Effect of the NaCl concentration on the coloring-temperature regions at [C18AA] = 25 mM and [TOAB] = 11.4 mM.

Figure 4 shows that the NaCl concentration significantly influences the coloring-temperature range, such as the appearance of the *T*_H_ region when the NaCl concentration is in the range of 0.75–1.5 mM or the decrease in both the *T*_L_ and *T*_H_ regions with the increase in the concentration. The increase in the NaCl concentration expanded the temperature range in the *T*_H_ region; however, it had an insignificant influence on the temperature range in the *T*_L_ region. Subsequently, the two coloring regions overlapped when [NaCl] > 1.5 mM. All emulsions exhibited red color in the *T*_L_ region (Figure 4) and showed a blue shift in the *T*_H_ region. A similar color change was observed in the overlapped region of [NaCl] > 1.5 mM, wherein the red color was retained at a lower temperature and then showed a blue shift upon heating. These visual observations are in agreement with the temperature-dependent *d* values (Figure S3). Accordingly, the thermal color variation at [NaCl] > 0.75 mM followed a similar trend irrespective of the NaCl concentration, and the only difference is whether the coloring region is in a continuous or discontinuous temperature range.

**Figure 5:**
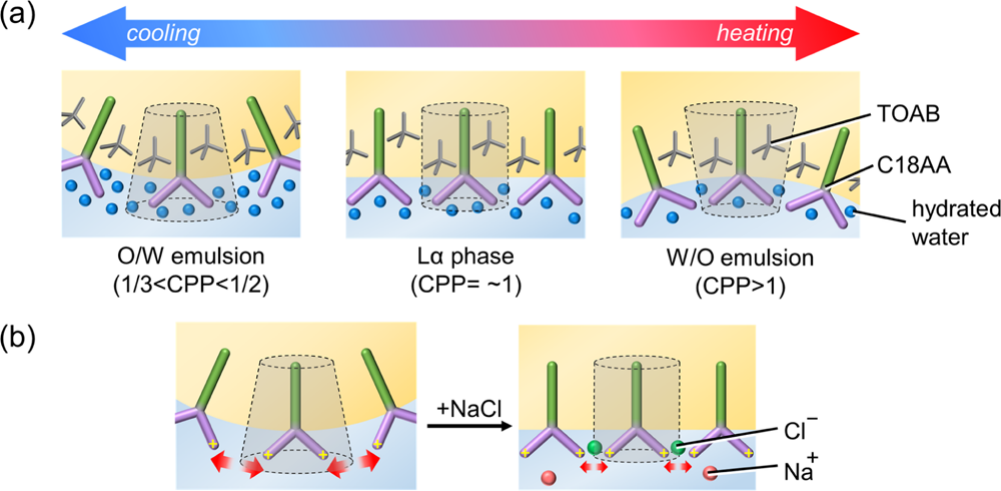
Schematic illustrations of the (a) thermal-induced phase inversion of the iridescent emulsion and the (b) NaCl-induced CPP value change of C18AA.

The lowering of the coloring-temperature range owing to the NaCl addition (Figure 4) can be explained by the critical packing parameter (CPP) concept proposed by Israelachvili.^43^ CPP is defined as *V*/*Al*, where *V* and *l* are the volume and length of the hydrophobic surfactant tail, respectively, and *A* is the optimal head group area. We first explain the thermal phase inversion of the current system using the CPP concept before discussing the effect of NaCl. According to a previous report on the present emulsion system without NaCl,^41^ the C18AA and TOAB emulsions undergo inversion from the O/W to the W/O emulsion phase upon heating through the lamellar phase, developing an iridescent color. Furthermore, the observed pH value for the aqueous solutions of C18AA was approximately 9.1, while the p*K*_a_ was approximately 9.5,^44^ indicating that the head groups of some C18AA molecules were positively charged. Increasing the temperature may cause a decrease in the number of water molecules hydrating the head groups of C18AA, thereby decreasing the *A* value and increasing the CPP value of C18AA. Because the suitable CPP values for the O/W emulsion, lamellar, and W/O emulsion phases are 1/2 < CPP <1/3, CPP = approximately 1, and CPP > 1, respectively,^45^ the increase in the CPP value results in the thermal-induced phase inversion (Figure 5a).

Applying this concept to the electrolyte concentration effect, the increase in the NaCl concentration may decrease the *A* value, increasing the CPP value of C18AA. This increase was attributed to the electrostatic shielding effect of the electrolyte ions that lowered the repulsion between the positively charged head groups of C18AA adsorbed on the water–toluene interface (Figure 5b). The increase in the CPP value decreases the CPP value increment to reach the lamellar phase, that is, it decreases the temperature increment required to reach the coloring-temperature regions. Consequently, the NaCl addition lowered the coloring-temperature regions.

### 3.3 Optical Observation of Emulsions in Coloring-Temperature Regions

Another characteristic feature of the NaCl system is the appearance of two coloring-temperature regions and the distinguishing color-changing features in the *T*_H_ region. The *T*_L_ region in Figure 4 is identical to the coloring region without NaCl; thus, it can be concluded that the *T*_L_ region is the lamellar phase.^41^ The *T*_H_ region is also considered the lamellar phase, and the *T*_L_ and *T*_H_ regions are practically identical phases because the onset in the *T*_H_ region developed a color similar to that in the *T*_L_ region, irrespective of the NaCl and C18AA concentrations. Furthermore, the estimation of the identical phases is consistent as the discontinuous boundary (non-coloring region) between the *T*_L_ and *T*_H_ regions disappeared at [NaCl] > 1.5 mM. Moreover, there was no distinction between the two emulsions in the *T*_L_ or *T*_H_ region.

**Figure 6:**
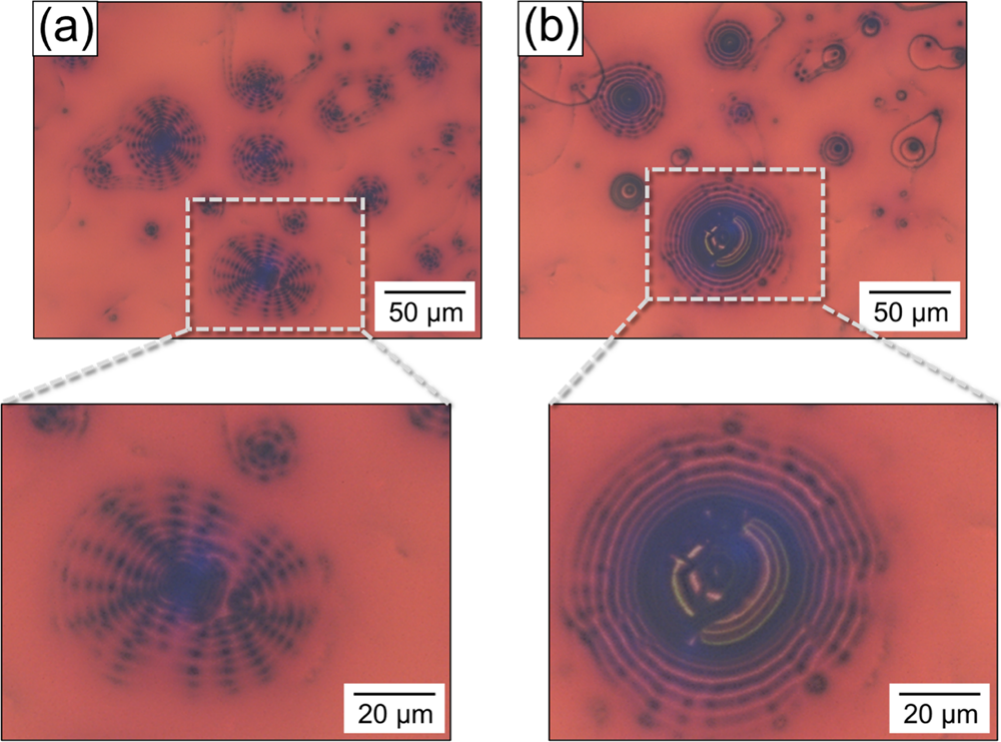
Optical reflection micrographs of the iridescent emulsion in the (a) *T*_L_ and (b) *T*_H_ regions; [C18AA] = 25 mM, [TOAB] = 11.4 mM, and [NaCl] = 1 mM.

To date, we have no reliable information with respect to the color change in the *T*_H_ region; subsequently, we observed the optical microscopic images of the emulsions. As shown in Figure 6a, there are black aggregates of a few micrometers surrounding a red background. Although the aggregates remained even at the onset of the *T*_H_ region, increasing the temperature led to partial melting of the aggregates and gradual morphological changes into a concentric circle pattern of red and black stripes (Figure 6b). The background color remained red; however, the red stripes of the aggregates became greenish. Further heating expanded the partially melted aggregates in all directions along with a blue shift of the background color. The aggregates disappeared at the end, and the background structural color became blue (Figure S4). Similar morphological changes in the aggregates and the blue shift were observed when [NaCl] = 2.5 mM where there was no boundary between the *T*_L_ and *T*_H_ regions (Figure S5).

**Figure 7:**
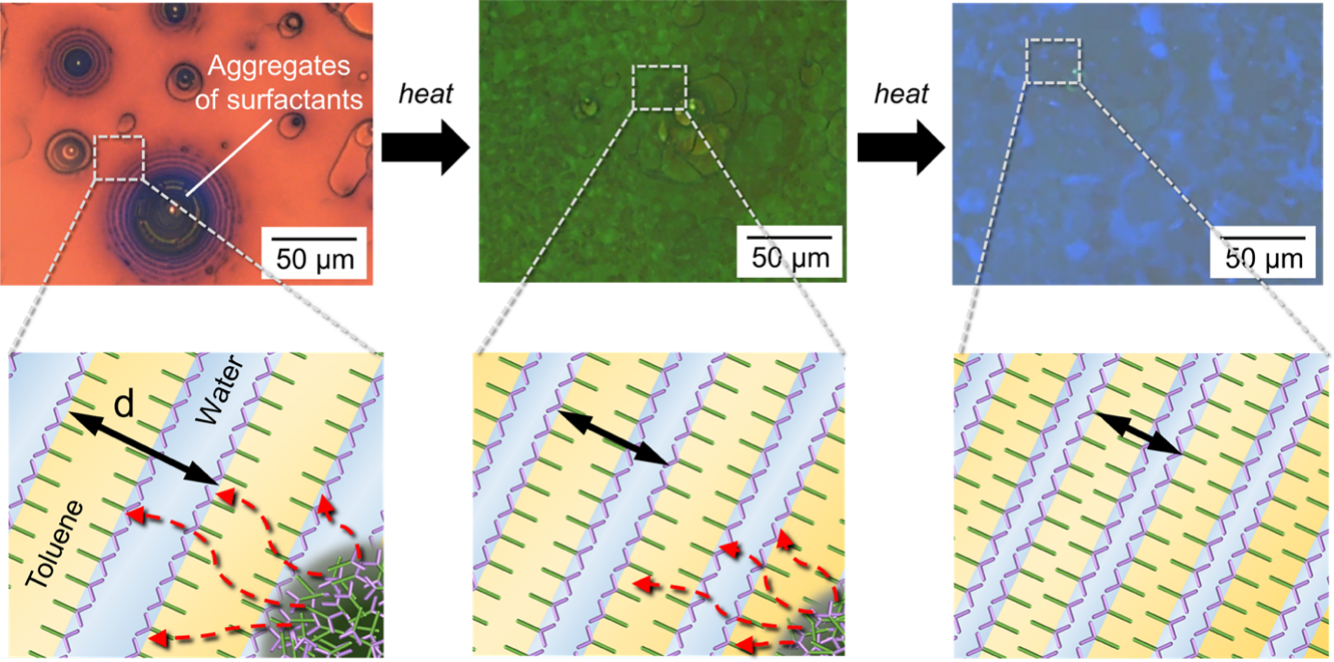
Schematic illustrations of the color-changing process in the *T*_H_ region upon heating.

The blue shift of the background structural color in the *T*_H_ region can be explained as follows: because the aggregates in the *T*_L_ region are a molecular assembly of C18AA or C18AA and TOAB, the melting of the aggregates upon heating may lead to the supply of C18AA to the background-coloring region. As shown in Figure 7, the increase in the C18AA concentration derived from the supply increases the interfacial area between water and toluene, leading to a decrease in the *d* value of the periodic layered structure. Furthermore, the appearance of a greenish color in the concentric circle aggregates before the background color change was attributed to the locally high C18AA concentration in the concentric-circle void of the aggregates, which was derived from the partial melting of the aggregates. Hence, the increase in the C18AA concentration led to the blue shift in the *T*_H_ region, which was induced by disassembling the aggregates upon heating.

### 3.4 Effects of the C18AA and TOAB Concentrations

**Figure 8:**
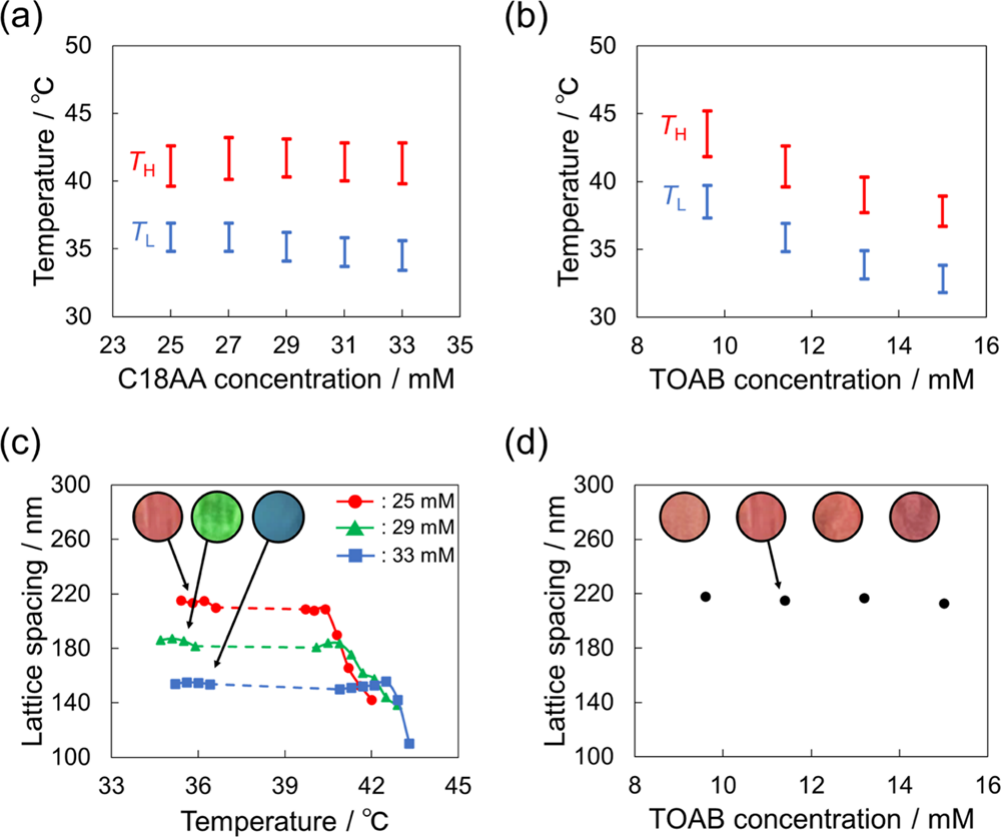
Effect of (a) C18AA and (b) TOAB concentrations on the coloring-temperature regions. (c) Temperature dependences of lattice spacing of the iridescent emulsion at [C18AA] = 25, 29, and 33 mM. (d) Effect of the TOAB concentration on the lattice spacing in the *T*_L_ region. The standard preparation condition of emulsions were [C18AA] = 25 mM, [TOAB] = 11.4 mM, and [NaCl] = 1 mM.

Our previous study reported that the color tone and coloring temperature of the emulsions with a single coloring-temperature region could be adjusted independently using the C18AA and TOAB concentrations, respectively. Herein, we investigated the effect of both concentrations on the two coloring-temperature regions of the current NaCl-containing emulsions. The two coloring-temperature regions were visible irrespective of the C18AA (Figure 8a) and TOAB (Figure 8b) concentrations. The coloring-temperature range of the regions was almost invariable with the C18AA concentration, while it decreased with the TOAB concentration. In contrast, the color tone was dependent on the C18AA concentration and not on the TOAB concentration (Figure 8c,d). As mentioned in Figure 7, the increase in the C18AA concentration leads to the blue shift of the structural color due to an increase in the interfacial area between water and toluene. Accordingly, increasing the C18AA varied the emulsion color from red to blue in the *T*_L_ region, and the onset color in the *T*_H_ region changed into the color corresponding to the *T*_L_ region. On the other hand, since TOAB does not directly adsorb on the interface, the TOAB concentration did not influence the interfacial area, leading to the constant color tone in Figure 8d. Consequently, the influence of their concentrations on the color and coloring temperature of the NaCl emulsion was similar to that of the previously reported emulsions without NaCl.^41^ Briefly, the color and coloring temperature of the NaCl-containing emulsions could be independently tuned using the C18AA and TOAB concentrations, respectively. Hence, a combination of these characteristic dependencies and color variations with temperature in the *T*_H_ region induces the ability of the emulsions to be all-liquid-type structural color materials that develop the desired color at a given temperature. The potential application of the present iridescent emulsions is thus a highly sensitive thermal sensor that can visually detect the precious temperature.

## 4 Conclusions

In this study, we successfully demonstrated that adding NaCl into the C18AA and TOAB emulsions produced all-liquid-type structural color materials embodying a thermo-responsive color change feature. An adequate NaCl concentration generated two coloring-temperature regions, the *T*_L_ and *T*_H_ regions, during the thermal phase inversion of the C18AA and TOAB emulsions. The emulsion color in the *T*_L_ region was constant while that in the *T*_H_ region showed a blue shift upon heating, indicating that the emulsions imparted the thermo-responsive color-changing feature after adding NaCl. The onset color in the *T*_H_ region was identical to that in the *T*_L_ region, and the color range in the *T*_H_ region was variable because the concentration of C18AA easily controlled the color in the *T*_L_ region. Furthermore, the TOAB and NaCl concentrations could regulate the coloring-temperature regions. Accordingly, the adjustability of the color and coloring-temperature regions of all-liquid-type emulsions can enable the realization of novel structural coloring materials that develop the desired color at any shape and temperature.

## Supporting Information Available

Photographs and the direct ultraviolet–visible reflection spectra of the iridescent emulsion at various incident angles at 36.5 °C and the corresponding plot of sin^2^ θ against λ^2^, photographs of the iridescent emulsion containing LiCl or NaBr at various temperatures, temperature dependences of lattice spacing of the iridescent emulsion in a 0.5 or 2.5 mM NaCl concentration, temperature variation of the optical reflection micrographs in the *T*_H_ region, and optical reflection micrographs of the iridescent emulsion in a 2.5 mM NaCl concentration (PDF)
