# Supplementary material for: Thermally Tunable Structural Coloration of Water/Surfactant/Oil Emulsions
Source: Langmuir. 2021 Dec 22;38(1):569–75. doi: 10.1021/acs.langmuir.1c03020 (PMC8757461; doi:10.1021/acs.langmuir.1c03020)
Supplement: Supplementary file 1 — la1c03020_si_001.pdf [file la1c03020_si_001.pdf]

SUPPORTING INFORMATION

Thermally Tunable Structural Coloration of Water/Surfactant/Oil Emulsions

Yuto Arai, Nayuta Yashiro, Yoshiro Imura, Ke-Hsuan Wang, and Takeshi Kawai*

Department of Industrial Chemistry, Tokyo University of Science,
1-3 Kagurazaka, Shinjuku-ku, Tokyo 162-8601, Japan

Email: kawai@ci.tus.ac.jp

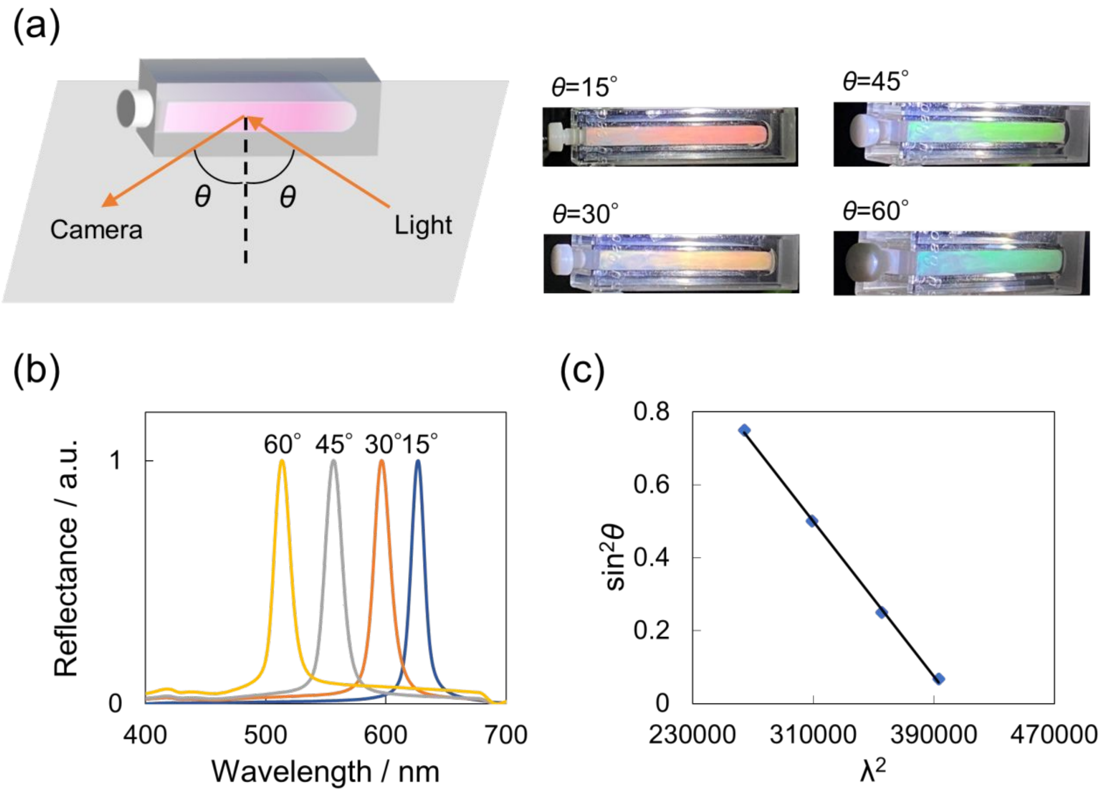

Figure S1. (a) Photographs and (b) the direct ultraviolet-visible reflection spectra of the iridescent emulsion at various incident angles at 36.5 °C. (c) The corresponding plot of $\sin^2\theta$ against λ^2, where θ and λ are the incident angle of light and wavelength of reflection peak, respectively.

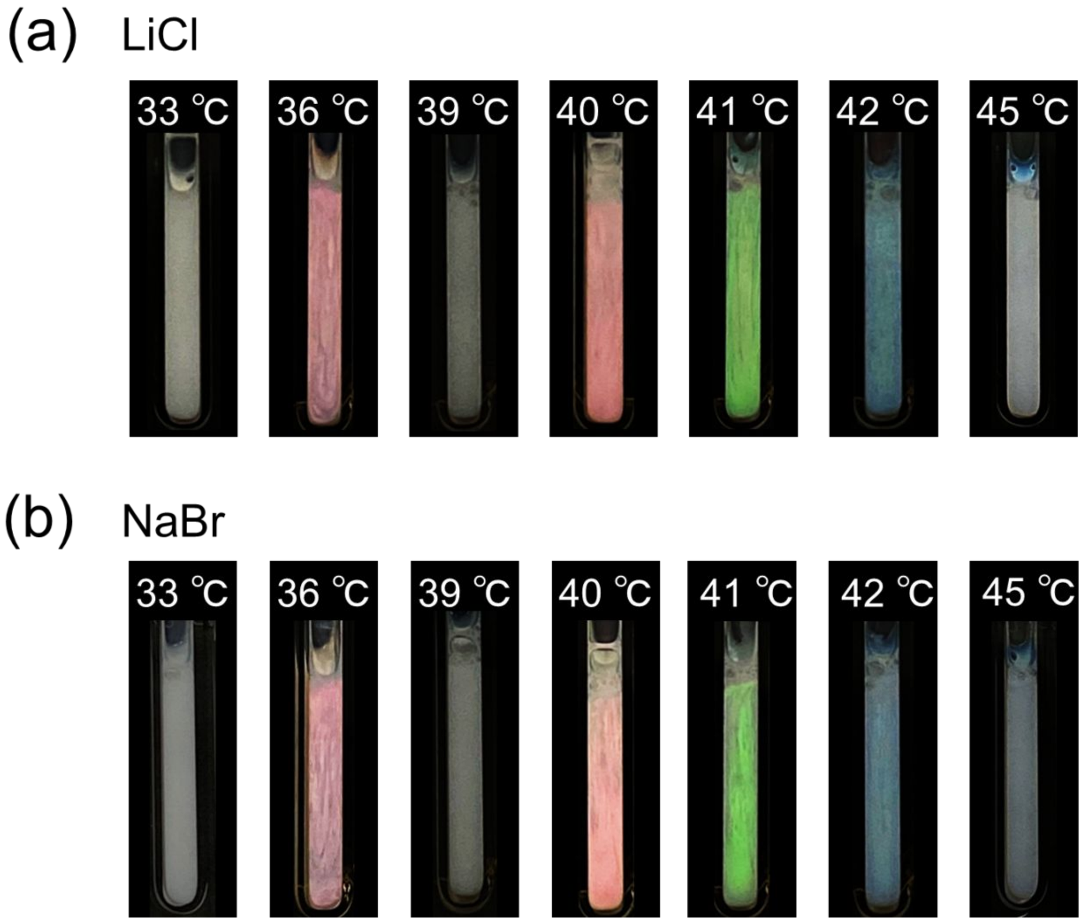

Figure S2. Photographs of the iridescent emulsion containing (a) LiCl and (b) NaBr electrolytes at various temperatures; [C18AA] = 25 mM and [tetraoctylammonium bromide] = 11.4 mM. The electrolyte concentration was 1 mM.

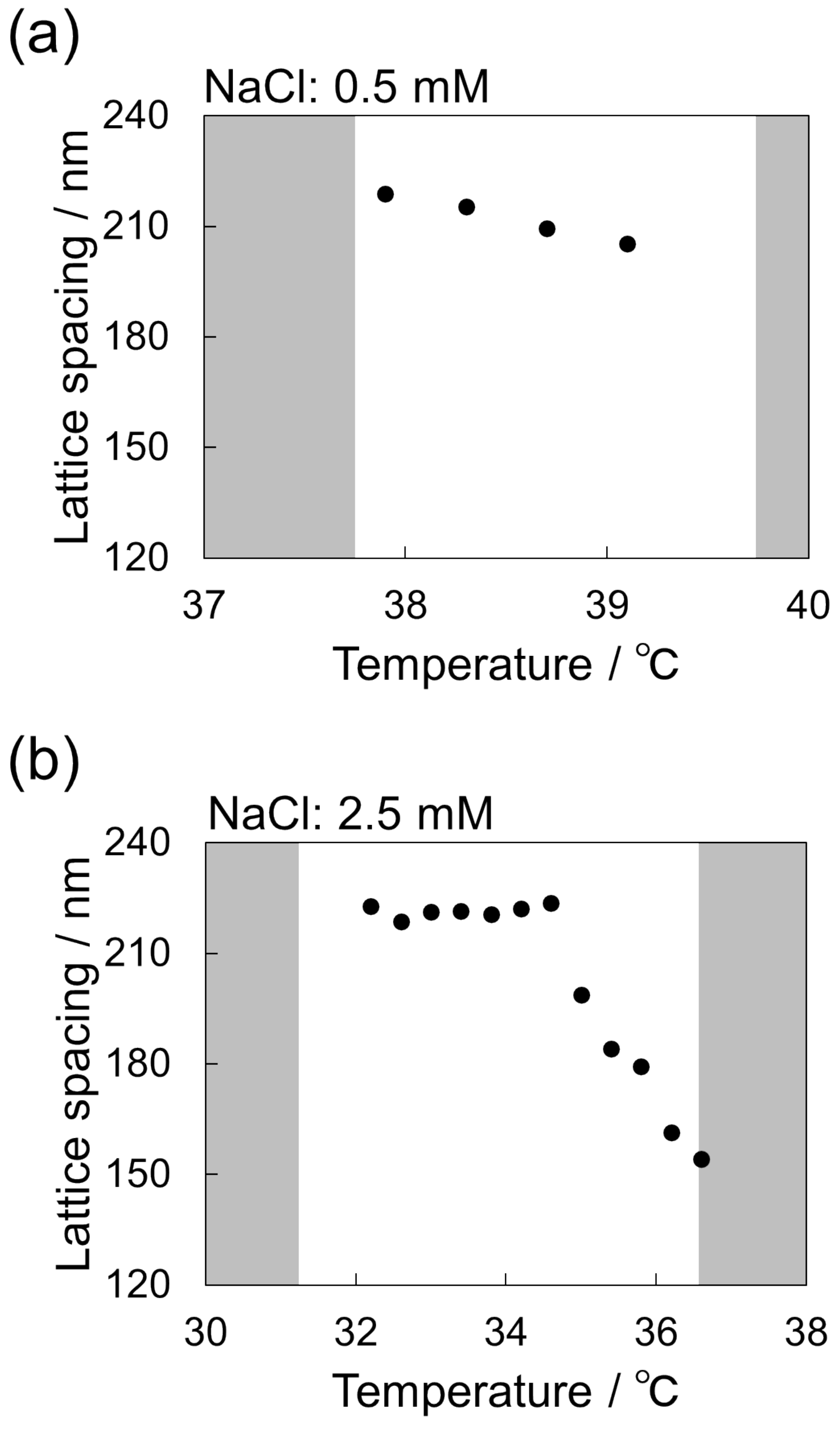

Figure S3. Temperature dependences of lattice spacing of the iridescent emulsion in (a) 0.5 mM and (b) 2.5 mM NaCl concentration; [C18AA] = 25 mM and [tetraoctylammonium bromide] = 11.4 mM.

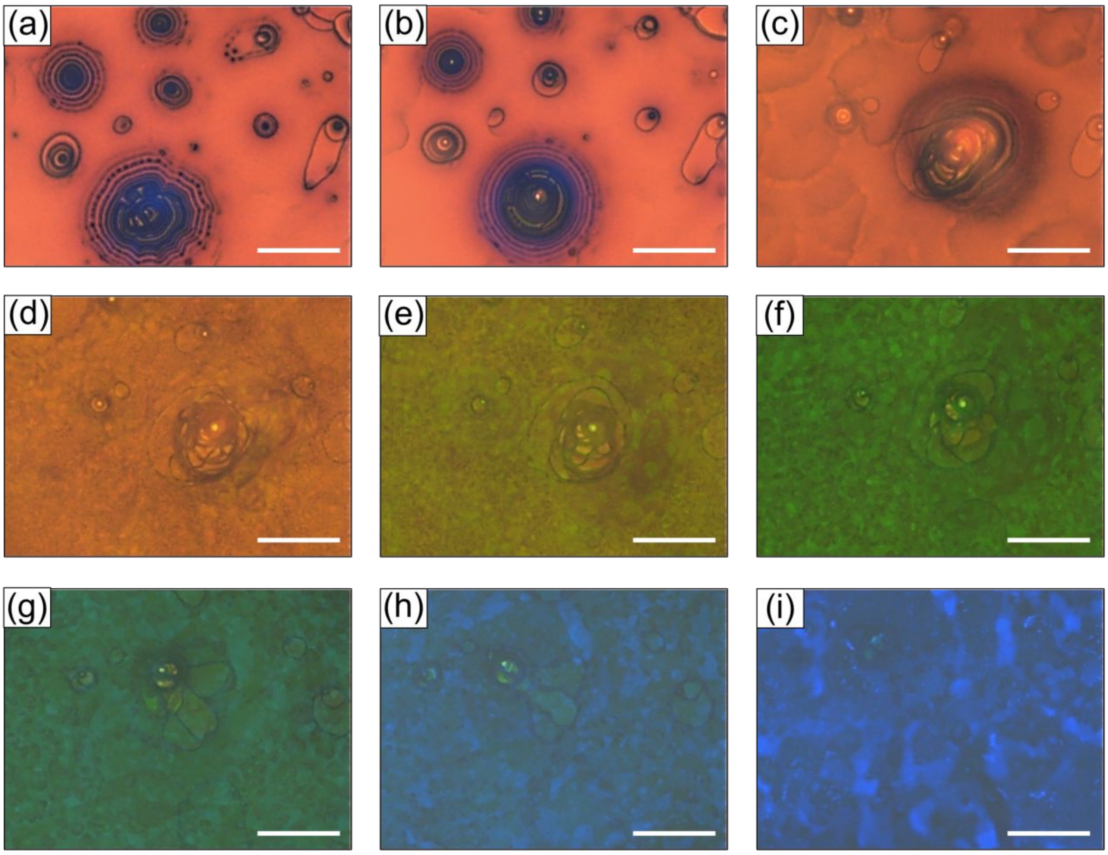

Figure S4. Temperature variation of the optical reflection micrographs in the T_H region. Scale bar is 50 μm. (a–i) Temperature changes from 40 ℃ to 42 ℃.

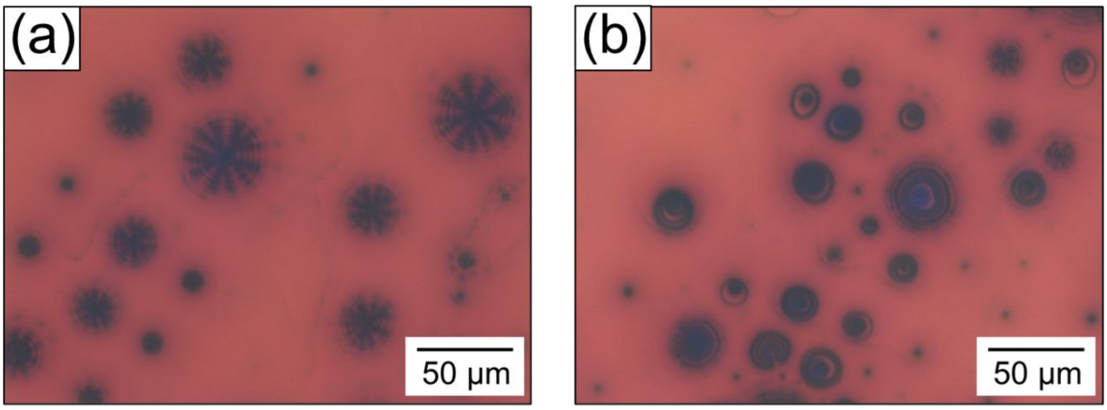

Figure S5. Optical reflection micrographs of the iridescent emulsion in the (a) T_L and (b) T_H regions; [C18AA] = 25 mM and [tetraoctylammonium bromide] = 11.4 mM. The observation was performed in 2.5 mM NaCl.
